# Sequencing of the IL6 gene in a case–control study of cerebral palsy in children

**DOI:** 10.1186/1471-2350-14-126

**Published:** 2013-12-07

**Authors:** Pouya Khankhanian, Sergio E Baranzini, Britt A Johnson, Lohith Madireddy, Dorothee Nickles, Lisa A Croen, Yvonne W Wu

**Affiliations:** 1 Department of Neurology, University of California, 675 Nelson Rising Lane, 94158, San Francisco, CA, USA; 2 Kaiser Permanente Division of Research, 2000 Broadway, 94612, Oakland, CA, USA; 3 Department of Pediatrics, University of California, 350 Parnassus, Ste 609, 94117, San Francisco, CA, USA

**Keywords:** Cerebral palsy, Sanger sequencing, IL-6, Interleukin-6, Haplotype

## Abstract

**Background:**

Cerebral palsy (CP) is a group of nonprogressive disorders of movement and posture caused by abnormal development of, or damage to, motor control centers of the brain. A single nucleotide polymorphism (SNP), rs1800795, in the promoter region of the *interleukin-6* (IL6) gene has been implicated in the pathogenesis of CP by mediating IL-6 protein levels in amniotic fluid and cord plasma and within brain lesions. This SNP has been associated with other neurological, vascular, and malignant processes as well, often as part of a haplotype block.

**Methods:**

To refine the regional genetic association with CP, we sequenced (Sanger) the IL6 gene and part of the promoter region in 250 infants with CP and 305 controls.

**Results:**

We identified a haplotype of 7 SNPs that includes rs1800795. In a recessive model of inheritance, the variant haplotype conferred greater risk (OR = 4.3, CI = [2.0-10.1], p = 0.00007) than did the lone variant at rs1800795 (OR = 2.5, CI = [1.4-4.6], p = 0.002). The risk haplotype contains one SNP (rs2069845, CI = [1.2-4.3], OR = 2.3, p = 0.009) that disrupts a methylation site.

**Conclusions:**

The risk haplotype identified in this study overlaps with previously identified haplotypes that include additional promoter SNPs. A risk haplotype at the IL6 gene likely confers risk to CP, and perhaps other diseases, via a multi-factorial mechanism.

## Background

Cerebral palsy (CP) is a group of nonprogressive motor impairment syndromes caused by lesions of the brain arising early in development. The motor disorders of CP are often accompanied by disturbances of sensation, perception, cognition, communication, and behavior; by epilepsy; and by secondary musculoskeletal problems [1]. A number of risk factors for the condition have been identified, including preterm birth, intrauterine infection, intrauterine growth restriction, perinatal stroke, and a sibling with CP [2-7]. How the fetus responds in the presence of these risk factors is influenced by its genetic makeup, with some genotypes creating susceptibility to cerebral damage [8,9]. Several rare genetic variants that cause Mendelian forms of CP have been described [10]; however, these do not account for the vast majority of CP cases.

*Interleukin-6* (IL6) is a cell signaling molecule that has been associated with over 20 diseases, including inflammatory, neurological, vascular, and malignant processes (Additional file 1: Table S1). The most studied single nucleotide polymorphism (SNP) in the IL6 gene is rs1800795 (−174G < C). This SNP lies within the promoter region and the C allele has been associated with either increases or decreases inIL6 levels in several tissues [11-14].

Both the IL-6 gene and IL-6 protein levels have been linked with increased risk of CP. Within the IL6 gene, the C allele at rs1800795 has been associated with increased risk of CP in large population studies [15-17]. Elevated cerebrospinal fluid IL-6levels have been associated with worse neurologic outcomes following hypoxic-ischemic encephalopathy [18]. Elevated IL-6 levels in umbilical cord plasma and amniotic fluid and within brain lesions have been associated with periventricular leukomalacia, a major risk factor for CP in preterm infants [19-22]. Higher cord blood IL-6 protein levels also predict cerebral lesions on MRI in preterm infants [23].

To our knowledge, no IL6 sequencing or haplotype studies have been performed to further evaluate the relationship between IL6 genotype and CP. Therefore, we performed Sanger sequencing of the IL6 gene in a nested case control study of CP among term infants to identifyIL6 haplotypes and mutations that predispose individuals to high risk of CP.

## Methods

### Study participants and samples

We studied 250 infants with CP and 305 randomly selected control infants born at < = 36 weeks gestation within the Kaiser Permanente Medical Care Program (KPMCP) during1991-2002, as previously described [17]. Briefly, we searched KPMCP records for physician diagnoses of “cerebral palsy,” “paresis,” “gait abnormality,” or “cerebral degeneration.” A single child neurologist (author YWW) reviewed medical records to confirm the diagnosis of CP. We defined CP as a non-progressive congenital motor dysfunction with examination findings of increased tone (spasticity, rigidity, dystonia) or choreoathetosis. Children with hypotonia, ataxia, myopathy, neural tube defect, genetic syndrome, and chromosomal anomaly were excluded. Infants with CP whose blood samples were unavailable for study (47), whose blood samples were taken after having received a blood transfusion (5), or whose blood samples were mislabeled (1), were excluded. A random sample of 305 infants from the study population with available blood samples comprised the control group of this study. All blood samples for DNA purification came from heel-stick dried blood collected in the neonatal period for purposes of newborn screening as part of the California Newborn Screening Program (NBS), unused portions of blood spots are stored in archives at the California Department of Public Health with standard procedure to avoid cross contamination [17]. For this study, punch biopsies of unused portions of blood spots were used for DNA extraction. We received a waiver of consent from the state of California IRB and the IRBs of the individual institutions. To prevent cross contamination between biopsies, the stainless punch was soaked in ethanol, then the ethanol-soaked punch was placed over open flame to burn off residual ethanol and DNA. DNA extraction and amplification was performed using QIAamp 96 DNA kits supplied by Qiagen (Chatsworth, CA), and RepliPHI Phi 29 Reagent Sets (Epicentre Technologies, Madison, WI) as previously described [17]. Ethnicity data was determined from chart review, using pre-defined categories within the KPMCP system: African American (26/22 = Cases/Controls), Asian (26/47), Hispanic (48/51), white (138/163), and others including mixed and unknown (12/22). Study procedures were approved by the institutional review boards at KPMCP, University of California San Francisco, and the California Committee for Protection of Human Subjects.

### Sanger sequencing

A combination of long and short amplicons was used to sequence 5000 base pairs covering the IL6 gene starting 300 bases upstream from the transcription start site. In total, 9 long amplicons and49 short amplicons were generated and later sequenced using standard di-deoxy terminator chemistry and capillary electrophoresis technology in an ABI 3730XL instrument (Applied Biosystems, Foster City, CA). Calls were made using Sequencher software (Gene Codes Corporation, Ann Arbor, MI). Genotype calls at important loci of interest were confirmed by manual calls made by two independent operators looking at peaks on an electropherogram using standard procedure. Any discrepant calls were labeled missing (Additional file 2: Table S2). SNP functionality was assessed using the poly-phen2 database [24] and the UCSC genome browser [25]. Hardy-Weinberg equilibrium was measured in controls from each population. In accordance with previous studies, the zero position is defined as the start of the transcribed portion of the gene, with numbers increasing in the direction of transcription, such that the +174 position aligns with rs1800795. Calls were made on the transcribed strand.

### Analysis

Linkage disequilibrium (LD) between the rs1800795 SNP and neighboring SNPs has been described in at least 9 studies, as detailed in Additional file 1: Table S1. The haplotype block of interest is defined differently in each of these studies, making it impossible to define a single consensus haplotype block of interest containing rs1800795. To resolve ambiguity regarding haplotype block definition in IL6, particularly the block containing rs1800795 (Additional file 1: Table S1), we began by investigating haplotype structure in healthy controls from each population. After reproducing the association between CP and rs1800795, we performed focused tests for association between CP and the haplotype containing rs1800795, followed by tests for association between CP and likely functional variants. Finally, we performed an unbiased search for association between CP and all other variants and haplotype blocks. For all association testing, the four populations were pooled; for association testing for common variants, population subgroup analysis was also performed. The R base package was used for statistical analysis except as stated. To elucidate the effect of known CP-associated SNPs and haplotypes, two simultaneous tests were performed. The effect of a single copy of variant allele was compared to wildtype using a Fisher’s exact test on the 2 × 2 table (heterozygote vs. homozygote wildtype), and the effect of two copies of the variant allele was measured using a Fisher’s exact test on a 2 × 2 table (homozygote variant vs. homozygote wildtype). This two-part test has the ability to capture a completely dominant model of inheritance (where both tests will be positive), a completely recessive model of inheritance (where only the second test is positive), a pure dose model of inheritance (where the effect size of the first test will be half the effect size of the second test), or any other model within the spectrum. Screening for other variants in IL6 that have not been previously described was performed as follows. For common variants, a “genotypic” test was performed on the 2 × 3 table of genotype versus case status, as well as an allele frequency test both using Plink software. For rare variants, the allele frequency test was performed, but the “genotypic” test was replaced by a “dominant” test on a 2 × 2 table of genotypes ((homozygous variant + heterozygote) vs. wildtype). Logistic regression was also performed, yielded similar results, and is not shown.

### Haplotype definition

Variants were classified into haplotype blocks in each of the four large populations as follows. Pair-wise linkage disequilibrium between variants was calculated using r^2^. Variants were re-ordered by unsupervised hierarchical clustering using 1-r^2^ for distance and the Ward [26] algorithm of agglomeration. Branches of the dendrogram defined groups of non-sequential SNPs were grouped into haplotype blocks with intra-haplotype r^2^ < 0.3 (Additional file 3: Figure S1). In most cases, long sequencing reads provided haplotype (phased genotype) information for any given individual. When phasing could not be determined by direct sequencing results, a maximum likelihood model was used to infer haplotypes. An individual’s genotype at the haplotype block is defined on each chromosome. A haplotype is called wildtype if all SNPs called within the haplotype are wildtype and at least half of the SNPs within the haplotype are called. A haplotype is called variant if all SNPs within the haplotype are variant and at least half of the SNPs within the haplotype are called. Hardy-Weinberg equilibrium for the haplotype was measured in controls from each population.

## Results and discussion

### Descriptive statistics: variants and haplotypes called

**Figure 1:**
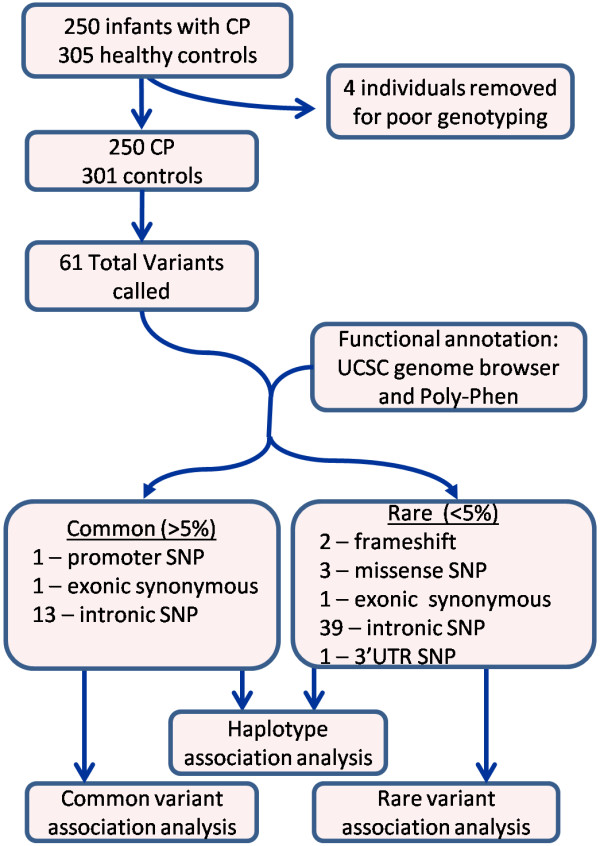
**Experimental design.** Experimental workflow: sample sizes, number and type of variants identified, and analyses performed.

In total, 61 variable loci were identified in the dataset (Figure 1, Additional file 4: Table S3). Fifteen of these were common polymorphisms in the population (defined by minor allele frequency (MAF) < 5% in controls), including previously described SNP rs1800795 (−174G < C) [17]. Uncommon variants (allele frequency less than 5%) included two frameshift mutations. One frameshift mutation at position 316 likely disrupts gene function by generating a stop codon near the 5′ end of the first exon of the gene, effectively removing nearly all protein domains. The other frameshift mutation at position 2494 is unlikely to be damaging because it occurs at the 3' end of the last exon of some transcript variants and does not affect other transcript variants.

Variants were grouped into haplotype blocks in each population using r^2^ as a measure of linkage disequilibrium, using Ward hierarchical clustering to re-order SNPs. The greatest linkage disequilibrium (LD) was seen in whites, followed closely by Hispanics and Asians, with African Americans showing the least LD (Additional file 3: Figure S1). In white controls, five haplotype blocks were identified with intra-block r^2^ < 0.3 and the inter-block r^2^ < 0.3 (Additional file 3: Figure S1). These five haplotype blocks included 18 of 61 variants. The rs1800795 SNP was included in a 7-SNP haplotype block: rs1800795 (−174), rs2069832 (615), rs2069833 (846), rs1474348 (1090), rs1474347 (1306), rs1554606 (1889), and rs2069845 (3331). In Hispanic controls, three haplotype blocks were identified, which included 13 variants. These blocks were identical to three of the five haplotype blocks identified in white controls. This includes the same 7-SNP haplotype block for rs1800795. In Asian controls, three haplotype blocks were identified including 11 variants. One of these blocks was again the same 7-SNP haplotype of rs1800795. Another block was identical to one found in Hispanics and whites. The third block found in Asians was a smaller version of the other block found in Hispanics and whites. Finally, in African Americans, five blocks were identified containing 12 SNPs. This time the rs1800795 SNP was part of a smaller 4-SNP haplotype, a subset of the 7-SNP haplotype found in the other three populations. The other four blocks found in African Americans included one block that was identified in all populations (1401 and 1431) and three novel blocks.

The 7-SNP block, which includes rs1800795 and was identified in whites, Hispanics, and Asians, includes an intronic SNP rs20698455 that disrupts a C_p_G methylation [27] along with 5 other intronic SNPs. The smaller 4-SNP block of rs1800795 identified in African Americans is a subset of the 7-SNP block but does not include the rs20698455 SNP. Overlap between the7-SNP haplotype and previously reported haplotypes is shown in red text in Additional file 1: Table S1.

### The rs1800795 haplotype

The rs1800795 SNP was identified as part of a 7-SNP haplotype block in whites, Hispanics, and Asians, and as part of a smaller 4-SNP block in African Americans. Among control infants, the variant haplotype allele was more common among whites (26% and 27% for the 7-SNP and 4-SNP haplotypes respectively) than among Hispanics (10% and 13%), Asians (7% and 7%), or African Americans (4% and 5%). There was no evidence of Hardy-Weinberg disequilibrium for either the haplotypes or for any of the SNPs in controls of each population.

The association between the 7-SNP haplotype and CP was stronger than the association seen between any individual SNP and CP, or between the 4-SNP haplotype and CP. Five of seven SNPs in this block showed significant association with CP with a recessive pattern of inheritance (Table 1). The odds ratio (OR) of association for homozygote versus wildtype at individual SNPs was at most 2.7. However, the OR for homozygotes for the variant at the full 7-SNP haplotype block variant was 4.3 (95% confidence interval (CI) = [2.0-10.1], Fisher exact test p = 0.00007, Bonferroni corrected p = 0.0004). The OR for homozygotes at the smaller 4-SNP haplotype was 3.0 (CI = [1.5-6.0], p = 0.0007, corrected p = 0.004). There was no evidence of increased risk for heterozygous carriers of any of these variant SNPs or for heterozygous carriers of the variant 4-SNP or 7-SNP haplotype.

**Table 1:** Genetic association with CP

**rs ID**	**Position**	**Homozygote vs. wt**	**OR**	**95% CI**	**P**	**Bonf**	
**rs1800795**	−174	C/C versus G/G	2.5	1.4-4.6	0.002	0.03	Promoter
**rs2069832**	615	A/A versus G/G	2.7	1.4-5.5	0.001	0.02	Intron
**rs2069833**	846	C/C versus T/T	2.4	1.3-4.6	0.004	0.05	Intron
**rs1474348**	1090	C/C versus G/G	1.5	0.6-3.6	0.33	1	Intron
**rs1474347**	1306	C/C versus A/A	1.8	0.8-3.6	0.1	0.9	Intron
**rs1554606**	1889	T/T versus G/G	2.5	1.5-4.5	0.0005	0.007	Intron
**rs2069845**	3331	G/G versus A/A	2.3	1.2-4.3	0.009	0.09	CpG site*
**4-SNP haplotype block**^**a**^			3.0	1.5-6.0	0.0007	0.004	
**7-SNP haplotype block**^**b**^			4.3	2.0-10.1	0.00007	0.0004	
**rs ID**	**Position**	**Homozygote vs. wt**	**OR**	**95% CI**	**P**	**Bonf**	
**rs1800795**	−174	C/G versus G/G	1.3	0.8-2.0	0.33	1	Promoter
**rs2069832**	615	A/G versus G/G	1.3	0.8-2.0	0.28	1	Intron
**rs2069833**	846	C/T versus T/T	1.4	0.9-2.1	0.19	1	Intron
**rs1474348**	1090	C/G versus G/G	1	0.6-1.7	1	1	Intron
**rs1474347**	1306	C/A versus A/A	0.7	0.3-1.8	0.5	1	Intron
**rs1554606**	1889	T/G versus G/G	1.1	0.8-1.7	0.5	1	Intron
**rs2069845**	3331	G/A versus A/A	1	0.5-2.3	1	1	CpG site*
**4-SNP haplotype block**^**a**^			1	0.6-1.6	1	1	
**7-SNP haplotype block**^**b**^			1	0.5-1.7	0.9	1	

*[27]; wt = wildtype; OR = odds ratio; CI = confidence interval; Bonf = Bonferroni correction for multiple hypothesis testing. For SNPs, correction was done for 14 total common SNPs tested.

^a^The 4-SNP block includes rs1800795, rs2069832, rs2069833, rs1474348. It was one of five haplotypes identified in African American controls. Bonferroni correction for five hypotheses tested.

^b^The 7-SNP block includes all SNPs listed in table. It was one of five haplotypes identified in white controls, one of three in Hispanic controls, and one of three in Asian controls. Bonferroni correction for 5 hypotheses tested.

**Figure 2:**
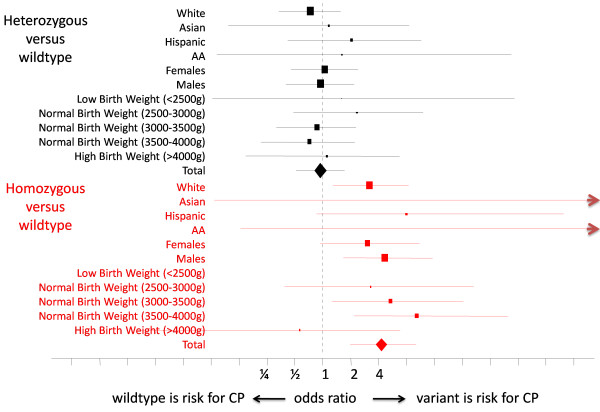
**Subgroup analysis.** Mean (boxes) and 95% confidence intervals (lines) for carriers of the variant haplotype by ethnicity, birth weight, and sex. Increased risk for homozygote carriers reached statistical significance in whites, Hispanics (one-sided only), males, and females (one-sided only). There was no risk associated with heterozygote carriers in any subgroup.

Population subgroup analysis of the 7-SNP haplotype block revealed a significant odds ratio for homozygous white (OR = 3.18, CI = [1.3-8.4], one-sided p = 0.007) and Hispanic infants (OR = 8.02, CI = [0.9-400], one-sided p = 0.045), with no evidence of increased risk for heterozygotes (Figure 2). Only two African American infants and one Asian infant were homozygotes; all three had CP. Population subgroup analysis of the 4-SNP haplotype block showed a similar pattern across populations, though effect sizes were proportionally reduced in relation to the 7-SNP haplotype (not shown). Subgroup analysis revealed significant odds ratios in males (OR = 4.6, CI = [1.6-15], one-sided p = 0.0006) and females (OR = 3.04, CI = [0.9-10.9], one-sided p = 0.03), and across body weights (there were no case or control homozygote variants with birth weight less than 2500 g).

### The frameshift mutation

As described above, one of the rare variants identified resulted in a frameshift at position 316 within the first exon and caused early protein truncation, thus warranting a focused analysis of this variant. Within this case–control dataset, the frameshift allele was found in four Asian infants (allele frequency = 2.5%), two Hispanic infants (allele frequency = 1%), three white infants (allele frequency = 0.6%), and one infant of other ethnicity. The lone homozygote was white. In total, eight of ten carriers of the frameshift allele were infants with CP (OR = 4.53, CI = [0.9-44], p-value = 0.053) including the lone homozygote carrier. Clinical characteristics of the eight infant frameshift carriers are shown in Table 2. Of note, three of eight carrier infants had perinatal stroke (versus 20.2% of non-carriers), including the lone homozygote carrier, though this result was not statistically significant. To control for possible confounding effects, individuals homozygous for the variant haplotype allele described above were excluded in the analysis of the frameshift mutation.

**Table 2:** Clinical characteristics of frameshift carriers

		**n**
Severity*	Moderate	2
	Severe	6
**Type of CP^**	Hemiparesis	4
	Quadriparesis	2
	Paraparesis	1
	Monoparesis	1
**Neuroimaging**	Perinatal Stroke	3
**(6CT, 1MRI)**	Global injury / volume loss	2
	No abnormalities	2
	No imaging	1
**Perinatal**	Emergent Caesarian	4
**Complications**	Preeclampsia	1
	Maternal temp < 100.5	1
	Meconium	1
	Neonatal seizures	2

*Moderate/severe = impaired function of most affected limb; mild = unimpaired function.

^All patients were diagnosed by a pediatric neurologist. Spasticity was present in all 8 patients; none had extra-pyramidal signs.

### Analysis of other variants

Finally, we carried out an unbiased analysis of the fifteen common variants identified in this population. When corrected for multiple hypotheses testing using the method of Bonferroni, we found no evidence of association (p < 0.05) with CP for any variants other than those described above. This was true for the entire dataset (Additional file 5: Table S4) and also for each population subgroup (not shown). Of the 46 rare variants, 20 were found in more than one individual. Unbiased analysis of these 20 rare variants using a dominant model revealed no significant association after correction for multiple testing (Additional file 6: Table S5). We deemed this analysis underpowered due to the small sample size and the large number of hypotheses tested.

## Conclusions

In this study, we found strong association of CP with an extended 7-SNP haplotype containing the functional promoter SNP rs1800795 in a recessive model of disease inheritance. We observed greater risk for homozygotes at the entire haplotype than for homozygotes at a smaller 4-SNP haplotype identified in African Americans, or for homozygotes at any individual locus. This suggests that there may be more than one functional locus within the 7-SNP haplotype, and that at least one functional locus may be outside of the smaller 4-SNP haplotype. In addition to the promoter function ofrs1800795 that is within the smaller 4-SNP haplotype, disruption of a methylation site at position rs2069845 [27] could be part of the mechanism conferring additional risk.

The variant rs1800795 has been implicated in various neurological, vascular, and malignant processes (Additional file 7). In these disease processes, it has often been implicated as part of a haplotype with (or implicated simultaneously with) other SNPs including many of the SNPs identified in this study (Additional file 1: Table S1). Two SNPs which have been previously found on haplotypes with rs1800795 include a rare missense SNP rs13306435(4220 T < A) [28] and an intron SNP rs2069840 [29]; neither showed evidence of association with CP in this study, although analysis of the rare missense SNP was likely underpowered. Other SNPs that have been previously found on haplotypes with rs1800795 but were outside the genotyping bounds of this study include possibly functional promoter SNPs(rs1800796 and rs1800797) [13] and less likely functional SNPs from the 5′ untranslated region (rs12700386, rs7802307, rs7802308, rs2069827) and the 3′ untranslated region (rs11766273, rs2069861, rs1818879). A study published after our analysis was complete [30] suggests that rs1800796-rs2069837 may be associated with risk in a subset of CP patients: Chinese males with spastic paraplegia. We found no association between rs2069837 and CP. However, our analysis was underpowered as this variant is rare in whites, Hispanics, and African Americans. The effect of the rs1800795 variant or the entire haplotype variant on the expression level of IL6 is not fully understood, with evidence for the CC genotype being associated with high or low expression in different tissues [11-14].

We found a trend for association of CP with a likely damaging rare frameshift mutation at position 316. Due to the rarity of this allele (frequency ~ 1%), a larger study would be needed to confirm this finding. Larger studies or meta-analyses can assess potential association with the other uncommon variants. However, such variants would be uncommon, even in CP patients, and therefore explain a small fraction of population risk.

There are a variety of common approaches to haplotype definition which have been used to describe IL6. Among other common methods (Additional file 1: Table S1), the method presented here is appealing because it achieves unequivocal haplotype boundaries (i.e. intra-haplotype r^2^ < 0.3, inter-haplotype r^2^ < 0.3 for any pair-wise comparison of SNPs). This method is not impaired by contiguous SNP inclusion criteria (i.e. it allows defined haplotypes to include non-contiguous SNPs), an impairment which would have made the discovery of this haplotype impossible in a sequencing dataset due to the high number of variants found. This method minimizes probability of winner’s curse by reducing the number of hypotheses tested to less than n (where n is the number of SNPs found), while other methods increase the number of tests to several fold higher than n. Other haplotype methods that seek independence rather than correlation can also achieve the same criteria, see for example [31]. A combination of both approaches (i.e. seeking an effect of a second haplotype while conditioning on the lead haplotype) is also possible, but did not yield significant results and was not shown. The method used in this current study is more likely to prove useful in a low-quality (high missing-ness) dataset where increasing signal-to-noise (by selecting multiple correlated measures) is more important.

Previous genetic studies of IL6 in CP have been limited to a handful of SNPs; sequencing presented several advantages. We have been able to fully describe the haplotype block of rs1800795 (to within the bounds of genotyping). Sequencing has also revealed a frameshift variant that causes early protein termination and absence of the four largest protein domains. Finally, sequencing allows us to rule out any other high-risk common variants (other than those discussed) within the boundaries of genotyping. A limitation of this study is the boundaries of genotyping. Functional promoter elements could be part of the mechanism conferring additional risk to CP. Another limitation was the low quality of the sequencing due in large part to low quantity of blood available in each sample, as this study “recycled” unused portions of blood samples after they had been used for routine newborn screening. To address this, we used manual calls made by 2 independent operators to confirm significant findings, discarding all calls that were not concordant. Thus, we sacrificed call rate for accuracy. Another limitation is the lack of confirmation of genotyping and lack of external replication, although it is reassuring that the main haplotype findings were significant independently in whites and Hispanics, as well as in males and females. The haplotype identified in this study, plus additional SNPs in the promoter region, may play a role in other diseases. Targeted genotyping of all haplotype SNPs in CP and other diseases using independent replication populations would be of interest. A genome-wide association study of CP may reveal additional genetic loci which predispose to this heterogeneous group of diseases.

## Competing interests

The authors have no competing interests to declare.

## Authors’ contributions

YWW, LAC, SEB, and PK conceived and designed the experiment. YWW and LAC performed chart review and selected samples. YWW, PK, BJ, LM, and DN performed data analysis. All authors read and approved the manuscript.

## Authors’ information

YWW is a professor in the Departments of Neurology and Pediatrics at the University of California, San Francisco (UCSF), and is supported by the National Institute of Health (NINDS K02 NS46688), and the United Cerebral Palsy Foundation (EH-005-03). SEB is a professor in the Department of Neurology at the UCSF and is a Harry Weaver Neuroscience Scholar of the National Multiple Sclerosis Society. LAC is a senior research scientist and directory of the Autism Research Program at Kaiser Permanente. The funding bodies played no role in the design, collection, analysis, interpretation of data, writing of the manuscript, or decision to publish the manuscript for presentation.

## Pre-publication history

The pre-publication history for this paper can be accessed here:

http://www.biomedcentral.com/1471-2350/14/126/prepub

## Supplementary Material

Additional file 1: Table S1 rs1800795 haplotype. A survey of published haplotype analyses of rs1800795. Click here for file

Additional file 2: Table S2 SNP-level missingness. Genotypes were labeled missing when consensus could not be reached by independent operators making genotype calls. Click here for file

Additional file 3: Figure S1 A - Linkage Disequilibrium. Haplotype blocks of r-squared in white controls. B - Linkage Disequilibrium. Haplotype blocks of r-squared in Hispanic controls. C - Linkage Disequilibrium. Haplotype blocks of r-squared in African American controls. D - Linkage Disequilibrium. Haplotype blocks of r-squared in Asian controls. Click here for file

Additional file 4: Table S3 Summary of variants identified. A total of 61 variants were observed in all samples including cases and controls. Click here for file

Additional file 5: Table S4 Unbiased analysis of common variants. No evidence for association outside of the rs1800795 haplotype block after correction for multiple hypothesis testing. Click here for file

Additional file 6: Table S5 Unbiased analysis of common variants. No evidence for association after correction for multiple hypothesis testing. Click here for file

Additional file 7 **rs1800795 and human disease.** A survey of published studies linking this SNP to human disease in the last decade. Click here for file
