# Supplementary figures and images for: Sequencing of the IL6 gene in a case–control study of cerebral palsy in children
Source: BMC Med Genet. 2013 Dec 7;14:126. doi: 10.1186/1471-2350-14-126 (PMC3881497; doi:10.1186/1471-2350-14-126)

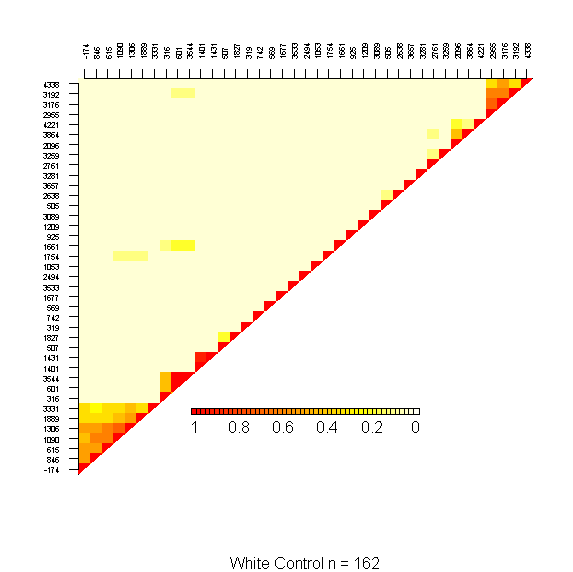

Supplement: Additional file 3: Figure S1 — A - Linkage Disequilibrium. Haplotype blocks of r-squared in white controls. B - Linkage Disequilibrium. Haplotype blocks of r-squared in Hispanic controls. C - Linkage Disequilibrium. Haplotype blocks of r-squared in African American controls. D - Linkage Disequilibrium. Haplotype blocks of r-squared in Asian controls. [file 1471-2350-14-126-S3.zip › 1167206147102043_Figure S1a.tiff]

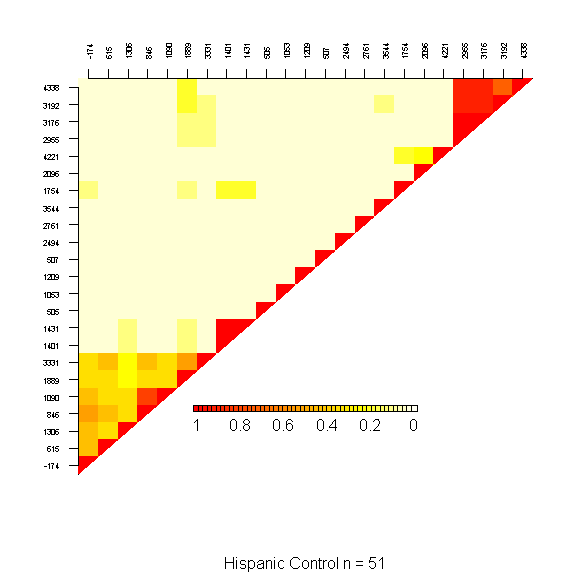

Supplement: Additional file 3: Figure S1 — A - Linkage Disequilibrium. Haplotype blocks of r-squared in white controls. B - Linkage Disequilibrium. Haplotype blocks of r-squared in Hispanic controls. C - Linkage Disequilibrium. Haplotype blocks of r-squared in African American controls. D - Linkage Disequilibrium. Haplotype blocks of r-squared in Asian controls. [file 1471-2350-14-126-S3.zip › 1167206147102043_Figure S1b.tiff]

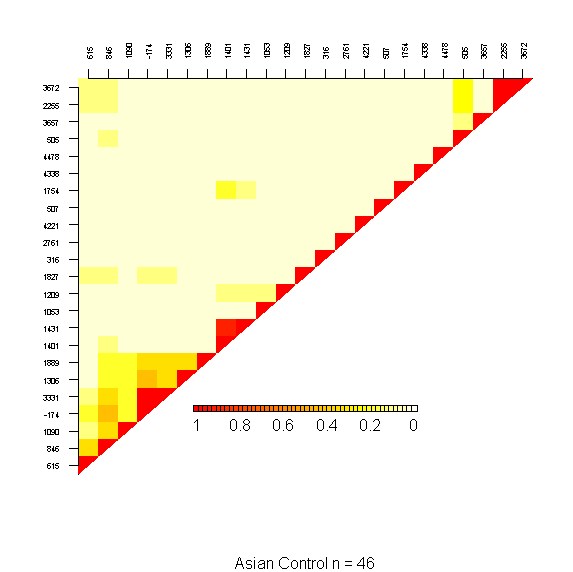

Supplement: Additional file 3: Figure S1 — A - Linkage Disequilibrium. Haplotype blocks of r-squared in white controls. B - Linkage Disequilibrium. Haplotype blocks of r-squared in Hispanic controls. C - Linkage Disequilibrium. Haplotype blocks of r-squared in African American controls. D - Linkage Disequilibrium. Haplotype blocks of r-squared in Asian controls. [file 1471-2350-14-126-S3.zip › 1167206147102043_Figure S1c.tiff]

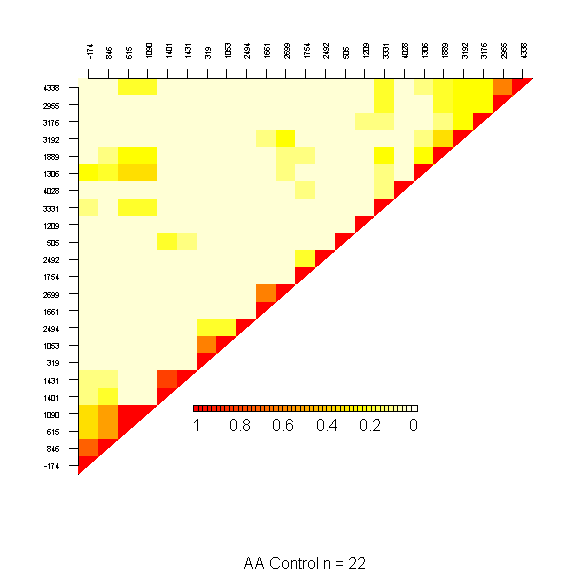

Supplement: Additional file 3: Figure S1 — A - Linkage Disequilibrium. Haplotype blocks of r-squared in white controls. B - Linkage Disequilibrium. Haplotype blocks of r-squared in Hispanic controls. C - Linkage Disequilibrium. Haplotype blocks of r-squared in African American controls. D - Linkage Disequilibrium. Haplotype blocks of r-squared in Asian controls. [file 1471-2350-14-126-S3.zip › 1167206147102043_Figure S1d.tiff]
