# Supplementary material for: Sequencing of the IL6 gene in a case–control study of cerebral palsy in children
Source: BMC Med Genet. 2013 Dec 7;14:126. doi: 10.1186/1471-2350-14-126 (PMC3881497; doi:10.1186/1471-2350-14-126)
Supplement: Additional file 7 — rs1800795 and human disease. A survey of published studies linking this SNP to human disease in the last decade. [file 1471-2350-14-126-S7.docx]

rs1800795 has been implicated in various neurological, vascular, and malignant processes as detailed below.

The presence of the G allele at rs1800795 was associated with increased risk of glioma[1], neuroblastoma[2], seizure frequency in patients with drug refractory epilepsy[3], severity of distal interphalangeal osteoarthritis[4], increased risk of disc degeneration (DD) in a Danish population[5] (as well as the G allele of rs1800797) and a Finnish population[6] (as part of a G-G-G haplotype on rs1800797-rs1800796-rs1800795), longer duration of disability due to sciatica[7] (as part of a G-G-G-A haplotype on rs1800797-rs1800796-rs1800795-rs13306435), and systemic sclerosis[8] (as part of a G-G-C haplotype at rs2069827-rs1800795-rs2069840). The G/G genotype has a neuroprotective role on hippocampus volumes[9], increases risk for Parkinson's Disease[10] and Schizophrenia[11], is associated with endothelial damage in rheumatoid arthritis[12], breast cancer[13-15] (as well as the G/G genotype at rs2069832), colon tumor mutations[16], and hyperlipidemia[17].

The C allele at rs1800795 has been associated with increased risk for multiple sclerosis in a Polish population[18], Celiac disease[19], stroke in women[20], post infectious irritable bowel syndrome[21], acute graft-vs.-host disease[22], type 2 leprosy reaction[23] (as part of a C-C haplotype at rs2069840-rs1800795, in addition to the A-C haplotype rs2069832-rs2069840), and hypertension[24]. The C allele[25] (as well as the C/C genotype at the -572 locus[26]) increases risk of Alzheimer's disease. The C/C genotype was overrepresented among subjects with high lytic titers in human herpesvirus-8 infection[27] and non-responders to rituximab in rheumatoid arthritis[28].

Some diseases show complex, sometimes conflicting association at rs1800795. In type II diabetes, the G/G genotype was associated with risk of diabetes in one study[29] but another study found the C/C genotype had increased mortality risk in dialysis patients[30]; a meta analysis found no association[31]. There is a complex association with adiposity, fat oxidation, body mass index, and power performance in athletes[17, 32-35]. Acute coronary syndrome has been associated with both the C allele[36-38] and the G allele[39] (as part of G-G-A-G-G haplotype at rs1800797-rs1800796-rs7802307-rs7802308-rs1800795). In one study, the C/C genotype was associated with reduced risk of depression[40], but another study associated the C/C and G/G genotypes with a greater risk[41].

The two alleles rs1800795 are canonical base pairs; therefore, the variant base is indistinguishable from the wildtype base unless the cis/trans strands have been identified[42]. Further, the two alleles occur in approximately equal frequency in many populations, so it is not possible to use allele frequency to determine the strand. Studies rarely identify the cis/trans strand explicitly. Studies which describe the polymorphism as -174G>C are assumed to be reporting the cis strand, as this is inherent in the chosen nomenclature. Array-based studies, including genome-wide studies (which always report the rs identifier "rs1800795"), will often report different strands depending on the processing algorithm, and these studies rarely publish cis/trans information.

# References

1. Brenner AV, Butler MA, Wang SS, Ruder AM, Rothman N, Schulte PA, Chanock SJ, Fine HA, Linet MS, Inskip PD: **Single-nucleotide polymorphisms in selected cytokine genes and risk of adult glioma**. *Carcinogenesis* 2007, **28**(12):2543-2547.

2. Lagmay JP, London WB, Gross TG, Termuhlen A, Sullivan N, Axel A, Mundy B, Ranalli M, Canner J, McGrady P *et al*: **Prognostic significance of interleukin-6 single nucleotide polymorphism genotypes in neuroblastoma: rs1800795 (promoter) and rs8192284 (receptor)**. *Clin Cancer Res* 2009, **15**(16):5234-5239.

3. Tiwari P, Dwivedi R, Mansoori N, Alam R, Chauhan UK, Tripathi M, Mukhopadhyay AK: **Do gene polymorphism in IL-1beta, TNF-alpha and IL-6 influence therapeutic response in patients with drug refractory epilepsy?** *Epilepsy Res* 2012, **101**(3):261-267.

4. Kamarainen OP, Solovieva S, Vehmas T, Luoma K, Riihimaki H, Ala-Kokko L, Mannikko M, Leino-Arjas P: **Common interleukin-6 promoter variants associate with the more severe forms of distal interphalangeal osteoarthritis**. *Arthritis Res Ther* 2008, **10**(1):R21.

5. Eskola PJ, Kjaer P, Sorensen JS, Okuloff A, Wedderkopp N, Daavittila I, Ala-Kokko L, Mannikko M, Karppinen J: **Gender difference in genetic association between IL1A variant and early lumbar disc degeneration: a three-year follow-up**. *Int J Mol Epidemiol Genet* 2012, **3**(3):195-204.

6. Kelempisioti A, Eskola PJ, Okuloff A, Karjalainen U, Takatalo J, Daavittila I, Niinimaki J, Sequeiros RB, Tervonen O, Solovieva S *et al*: **Genetic susceptibility of intervertebral disc degeneration among young Finnish adults**. *BMC Med Genet* 2011, **12**:153.

7. Karppinen J, Daavittila I, Noponen N, Haapea M, Taimela S, Vanharanta H, Ala-Kokko L, Mannikko M: **Is the interleukin-6 haplotype a prognostic factor for sciatica?** *Eur J Pain* 2008, **12**(8):1018-1025.

8. Cenit MC, Simeon CP, Vonk MC, Callejas-Rubio JL, Espinosa G, Carreira P, Blanco FJ, Narvaez J, Tolosa C, Roman-Ivorra JA *et al*: **Influence of the IL6 Gene in Susceptibility to Systemic Sclerosis**. *J Rheumatol* 2012, **39**(12):2294-2302.

9. Baune BT, Konrad C, Grotegerd D, Suslow T, Birosova E, Ohrmann P, Bauer J, Arolt V, Heindel W, Domschke K *et al*: **Interleukin-6 gene (IL-6): a possible role in brain morphology in the healthy adult brain**. *J Neuroinflammation* 2012, **9**:125.

10. San Luciano M, Ozelius L, Lipton RB, Raymond D, Bressman SB, Saunders-Pullman R: **Gender differences in the IL6 -174G>C and ESR2 1730G>A polymorphisms and the risk of Parkinson's disease**. *Neurosci Lett* 2012, **506**(2):312-316.

11. Zakharyan R, Petrek M, Arakelyan A, Mrazek F, Atshemyan S, Boyajyan A: **Interleukin-6 promoter polymorphism and plasma levels in patients with schizophrenia**. *Tissue Antigens* 2012, **80**(2):136-142.

12. Palomino-Morales R, Gonzalez-Juanatey C, Vazquez-Rodriguez TR, Miranda-Filloy JA, Llorca J, Martin J, Gonzalez-Gay MA: **Interleukin-6 gene -174 promoter polymorphism is associated with endothelial dysfunction but not with disease susceptibility in patients with rheumatoid arthritis**. *Clin Exp Rheumatol* 2009, **27**(6):964-970.

13. Slattery ML, Curtin K, Sweeney C, Wolff RK, Baumgartner RN, Baumgartner KB, Giuliano AR, Byers T: **Modifying effects of IL-6 polymorphisms on body size-associated breast cancer risk**. *Obesity (Silver Spring)* 2008, **16**(2):339-347.

14. Slattery ML, Curtin K, Giuliano AR, Sweeney C, Baumgartner R, Edwards S, Wolff RK, Baumgartner KB, Byers T: **Active and passive smoking, IL6, ESR1, and breast cancer risk**. *Breast Cancer Res Treat* 2008, **109**(1):101-111.

15. Pooja S, Chaudhary P, Nayak LV, Rajender S, Saini KS, Deol D, Kumar S, Bid HK, Konwar R: **Polymorphic variations in IL-1beta, IL-6 and IL-10 genes, their circulating serum levels and breast cancer risk in Indian women**. *Cytokine* 2012, **60**(1):122-128.

16. Slattery ML, Wolff RK, Curtin K, Fitzpatrick F, Herrick J, Potter JD, Caan BJ, Samowitz WS: **Colon tumor mutations and epigenetic changes associated with genetic polymorphism: insight into disease pathways**. *Mutat Res* 2009, **660**(1-2):12-21.

17. Riikola A, Sipila K, Kahonen M, Jula A, Nieminen MS, Moilanen L, Kesaniemi YA, Lehtimaki T, Hulkkonen J: **Interleukin-6 promoter polymorphism and cardiovascular risk factors: the Health 2000 Survey**. *Atherosclerosis* 2009, **207**(2):466-470.

18. Mirowska-Guzel D, Gromadzka G, Mach A, Czlonkowski A, Czlonkowska A: **Association of IL1A, IL1B, ILRN, IL6, IL10 and TNF-alpha polymorphisms with risk and clinical course of multiple sclerosis in a Polish population**. *J Neuroimmunol* 2011, **236**(1-2):87-92.

19. Dema B, Martinez A, Fernandez-Arquero M, Maluenda C, Polanco I, Figueredo MA, de la Concha EG, Urcelay E, Nunez C: **The IL6-174G/C polymorphism is associated with celiac disease susceptibility in girls**. *Hum Immunol* 2009, **70**(3):191-194.

20. Cole JW, Brown DW, Giles WH, Stine OC, O'Connell JR, Mitchell BD, Sorkin JD, Wozniak MA, Stern BJ, Sparks MJ *et al*: **Ischemic stroke risk, smoking, and the genetics of inflammation in a biracial population: the stroke prevention in young women study**. *Thromb J* 2008, **6**:11.

21. Villani AC, Lemire M, Thabane M, Belisle A, Geneau G, Garg AX, Clark WF, Moayyedi P, Collins SM, Franchimont D *et al*: **Genetic risk factors for post-infectious irritable bowel syndrome following a waterborne outbreak of gastroenteritis**. *Gastroenterology* 2010, **138**(4):1502-1513.

22. Chien JW, Zhang XC, Fan W, Wang H, Zhao LP, Martin PJ, Storer BE, Boeckh M, Warren EH, Hansen JA: **Evaluation of published single nucleotide polymorphisms associated with acute GVHD**. *Blood* 2012, **119**(22):5311-5319.

23. Sousa AL, Fava VM, Sampaio LH, Martelli CM, Costa MB, Mira MT, Stefani MM: **Genetic and immunological evidence implicates interleukin 6 as a susceptibility gene for leprosy type 2 reaction**. *J Infect Dis* 2012, **205**(9):1417-1424.

24. Conen D, Cheng S, Steiner LL, Buring JE, Ridker PM, Zee RY: **Association of 77 polymorphisms in 52 candidate genes with blood pressure progression and incident hypertension: the Women's Genome Health Study**. *J Hypertens* 2009, **27**(3):476-483.

25. Mansoori N, Tripathi M, Luthra K, Alam R, Lakshmy R, Sharma S, Arulselvi S, Parveen S, Mukhopadhyay AK: **MTHFR (677 and 1298) and IL-6-174 G/C genes in pathogenesis of Alzheimer's and vascular dementia and their epistatic interaction**. *Neurobiol Aging* 2012, **33**(5):1003 e1001-1008.

26. Wang M, Jia J: **The interleukin-6 gene -572C/G promoter polymorphism modifies Alzheimer's risk in APOE epsilon 4 carriers**. *Neurosci Lett* 2010, **482**(3):260-263.

27. Brown EE, Fallin MD, Goedert JJ, Hutchinson A, Vitale F, Lauria C, Giuliani M, Marshall V, Mbisa G, Serraino D *et al*: **Host immunogenetics and control of human herpesvirus-8 infection**. *J Infect Dis* 2006, **193**(8):1054-1062.

28. Robledo G, Davila-Fajardo CL, Marquez A, Ortego-Centeno N, Callejas Rubio JL, de Ramon Garrido E, Sanchez-Roman J, Garcia-Hernandez FJ, Rios-Fernandez R, Gonzalez-Escribano MF *et al*: **Association between -174 interleukin-6 gene polymorphism and biological response to rituximab in several systemic autoimmune diseases**. *DNA Cell Biol* 2012, **31**(9):1486-1491.

29. Huth C, Heid IM, Vollmert C, Gieger C, Grallert H, Wolford JK, Langer B, Thorand B, Klopp N, Hamid YH *et al*: **IL6 gene promoter polymorphisms and type 2 diabetes: joint analysis of individual participants' data from 21 studies**. *Diabetes* 2006, **55**(10):2915-2921.

30. Verduijn M, Marechal C, Coester AM, Sampimon DE, Boeschoten EW, Dekker FW, Goffin E, Krediet RT, Devuyst O: **The -174G/C variant of IL6 as risk factor for mortality and technique failure in a large cohort of peritoneal dialysis patients**. *Nephrol Dial Transplant* 2012, **27**(9):3516-3523.

31. Qi L, van Dam RM, Meigs JB, Manson JE, Hunter D, Hu FB: **Genetic variation in IL6 gene and type 2 diabetes: tagging-SNP haplotype analysis in large-scale case-control study and meta-analysis**. *Hum Mol Genet* 2006, **15**(11):1914-1920.

32. Corpeleijn E, Petersen L, Holst C, Saris WH, Astrup A, Langin D, MacDonald I, Martinez JA, Oppert JM, Polak J *et al*: **Obesity-related polymorphisms and their associations with the ability to regulate fat oxidation in obese Europeans: the NUGENOB study**. *Obesity (Silver Spring)* 2010, **18**(7):1369-1377.

33. Qi L, Zhang C, van Dam RM, Hu FB: **Interleukin-6 genetic variability and adiposity: associations in two prospective cohorts and systematic review in 26,944 individuals**. *J Clin Endocrinol Metab* 2007, **92**(9):3618-3625.

34. Ruiz JR, Buxens A, Artieda M, Arteta D, Santiago C, Rodriguez-Romo G, Lao JI, Gomez-Gallego F, Lucia A: **The -174 G/C polymorphism of the IL6 gene is associated with elite power performance**. *J Sci Med Sport* 2010, **13**(5):549-553.

35. Tabassum R, Mahendran Y, Dwivedi OP, Chauhan G, Ghosh S, Marwaha RK, Tandon N, Bharadwaj D: **Common variants of IL6, LEPR, and PBEF1 are associated with obesity in Indian children**. *Diabetes* 2012, **61**(3):626-631.

36. Babu BM, Reddy BP, Priya VH, Munshi A, Rani HS, Latha GS, Rao VD, Jyothy A: **Cytokine gene polymorphisms in the susceptibility to acute coronary syndrome**. *Genet Test Mol Biomarkers* 2012, **16**(5):359-365.

37. Lima-Neto LG, Hirata RD, Luchessi AD, Silbiger VN, Stephano MA, Sampaio MF, Armaganijan D, Hirata MH: **CD14 and IL6 polymorphisms are associated with a pro-atherogenic profile in young adults with acute myocardial infarction**. *J Thromb Thrombolysis* 2012.

38. Ljungman P, Bellander T, Nyberg F, Lampa E, Jacquemin B, Kolz M, Lanki T, Mitropoulos J, Muller M, Picciotto S *et al*: **DNA variants, plasma levels and variability of interleukin-6 in myocardial infarction survivors: results from the AIRGENE study**. *Thromb Res* 2009, **124**(1):57-64.

39. Maitra A, Shanker J, Dash D, John S, Sannappa PR, Rao VS, Ramanna JK, Kakkar VV: **Polymorphisms in the IL6 gene in Asian Indian families with premature coronary artery disease--the Indian Atherosclerosis Research Study**. *Thromb Haemost* 2008, **99**(5):944-950.

40. Bull SJ, Huezo-Diaz P, Binder EB, Cubells JF, Ranjith G, Maddock C, Miyazaki C, Alexander N, Hotopf M, Cleare AJ *et al*: **Functional polymorphisms in the interleukin-6 and serotonin transporter genes, and depression and fatigue induced by interferon-alpha and ribavirin treatment**. *Mol Psychiatry* 2009, **14**(12):1095-1104.

41. Roetker NS, Yonker JA, Lee C, Chang V, Basson JJ, Roan CL, Hauser TS, Hauser RM, Atwood CS: **Multigene interactions and the prediction of depression in the Wisconsin Longitudinal Study**. *BMJ Open* 2012, **2**(4).

42. Sand PG: **A lesson not learned: allele misassignment**. *Behav Brain Funct* 2007, **3**:65.
